# Mechanical Response and Fracture Mechanisms of ψ-Graphene Containing Elliptical Defects: Effects of Aspect Ratio and Orientation

**DOI:** 10.3390/nano16181164

**Published:** 2026-09-16

**Authors:** Xu Chang, Huichao Li, Shuanghua Liang

**Affiliations:** 1 School of Computer Engineering, Jiangsu University of Architectural Technology, Xuzhou 221116, China; liangshuanghua@jsjzi.edu.cn; 2 School of Materials and Physics, China University of Mining and Technology, Xuzhou 221116, China

**Keywords:** ψ-graphene, molecular dynamics, mechanical properties, elliptical defects

## Abstract

Using molecular dynamics (MD) simulations, we systematically investigate the mechanical response and failure mechanisms of ψ-graphene containing elliptical nanoholes. The mechanical reliability of ψ-graphene is significantly influenced by the complex interplay between defect geometry and lattice anisotropy. Our findings reveal that for loading along the *x*-axis, mechanical properties are severely degraded at an aspect ratio (*AR*) of 1 but unexpectedly recover as the defect becomes more elongated (*AR* = 1 to 8). In contrast, loading along the *y*-axis leads to a persistent degradation of the mechanical properties for all *AR* values. Furthermore, we have investigated the influence of the elliptical defect’s orientation on the mechanical properties relative to the tensile axis. The results reveal a pronounced orientation-dependent anisotropy; notably, the mechanical performance reaches its minimum at a critical tilt angle of 75° rather than the expected 90°. This anomalous behavior is driven by a mixed-mode (shear-tension) failure mechanism, where the elliptical tips align with the lattice’s intrinsic failure planes, significantly lowering the energy barrier for bond rupture. These results provide a new perspective for understanding and modulating the mechanical properties of ψ-graphene, serving as quantitative guidance for the potential application of carbon-based two-dimensional materials in nano-devices.

## 1. Introduction

Since the successful isolation of graphene, two-dimensional (2D) materials have attracted extensive attention due to their exceptional electronic, mechanical, and thermal properties [1,2,3,4]. In recent years, researchers have shifted their focus toward non-hexagonal carbon allotropes to explore properties beyond those of honeycomb lattices. Fan et al. synthesized an ultraflat biphenylene network with periodically arranged nonhexagonal rings, demonstrating its intrinsic metallic nature [5]. Density functional theory and AIMD simulations reveal that Penta-Hexa-Octa-Tetra-Hepta (PHOTH) graphene is a metallic allotrope with exceptional structural stability, capable of maintaining its integrity at temperatures as high as 1000 K [6]. A novel 2D carbon allotrope, Dodecanophene, has been recently proposed by Lima et al., demonstrating an invariant Dirac cone that preserves its electronic features even under intense uniaxial deformation [7]. Ajori and his coworker have investigated the mechanical properties and fracture behavior of defective penta-graphene, and found that the mechanical response can be tuned through defect engineering to transition it from having a negative Poisson’s ratio behavior to a conventional material [8].

As a planar carbon allotrope composed of pentagonal and hexagonal rings, ψ-graphene has emerged as a compelling candidate for next-generation nano-devices [9]. Characterized by its metallic nature and unique structural symmetry, ψ-graphene exhibits significant mechanical anisotropy and high thermal stability, making it a subject of intense scientific interest [9]. Computational simulations suggested that its unique structural arrangement not only provides it with exceptional electronic characteristics but also highlights its potential for hydrogen storage [10]. Ref. [11] also revealed that the mechanical behavior of hydrogenated ψ-graphene is closely tied to the density and pattern of hydrogenation, providing an important basis for tuning the properties of various graphene-like materials [11]. Non-Equilibrium Molecular Dynamics (NEMD) simulations were utilized by Tao et al. to explore the mechanical properties of a multi-layered heterostructure comprising ψ-graphene and various 2D graphyne layers [12]. It was shown that defect percentage, dimension, and temperature significantly influence the Young’s modulus and toughness of these innovative nanostructures.

However, in practical applications, 2D materials are inevitably subject to structural defects such as vacancies, cracks, and nanoholes, which are often introduced during synthesis or device fabrication. These defects act as sites of stress concentration and can lead to a catastrophic reduction in mechanical integrity [13,14,15,16]. In Ref. [17], utilizing MD simulations, Zheng et al. have systematically investigated the stress field characteristics and structural responses of defective graphene sheets under uniaxial tensile loading. They have found that the stress distribution of the material is closely correlated with the size and shape of the defects. For example, their results have demonstrated that, while keeping the area of the rectangular defect constant, varying the aspect ratio can effectively tune the mechanical properties of graphene [17]. Similarly, by employing MD simulations, Wang et al. have established uniaxial tension models of single-layer graphene containing pores and three types of grain boundaries to investigate the coupled effects of structural defects on fracture strength, revealing a competitive mechanism between the intrinsic strength of graphene grain boundaries and the pore-induced fracture strength [18]. Recent MD simulations were used to examine the effects of localized holes (circular, square, diamond, and triangular) and random vacancies on the mechanical properties of ψ-graphene, and found that while defects generally reduce the elastic modulus, they unexpectedly increase the rupture strain [19].

Crucially, beyond naturally occurring defects, rapid experimental advances have enabled the precise sculpting of tailored defect geometries in 2D membranes. Controlled techniques, including TEM/STEM focused electron beam nanosculpting [20,21] and quantum optical lithography [22], allow for the rapid patterning of stable nanoscale pores, high-aspect-ratio slits (down to sub-2 nm widths), and complex feature geometries in monolayer and heterostructured sheets. These experimental breakthroughs demonstrate that fabricating directional, anisotropic elliptical defects or slits has become technically feasible at the nanoscale, underscoring the practical need to probe how these geometric features govern localized failure mechanisms. To date, while localized voids (such as circular, square, and triangular holes) have been widely explored in graphene and other graphene-like systems [13,14,15,16,17,18,19], the intricate mechanisms governing lattice-defect interactions in topologically complex, non-hexagonal allotropes remain poorly understood. In existing literature, expanding a standard void inevitably alters both its sharpness and the total vacancy concentration simultaneously, making it difficult to isolate their respective effects. To achieve geometric decoupling and align with experimentally realizable defect geometries, we introduce elliptical nanoholes in ψ-graphene, whose mechanical response to anisotropic elliptical defects has not been systematically investigated. Unlike conventional circular defects, elliptical defects introduce two additional degrees of freedom: the aspect ratio (*AR*, defined as the ratio of the semi-major axis to the semi-minor axis) and the tilt angle (*θ*, defined as the angle between the major axis of the ellipse and the horizontal loading direction). Specifically, we adopt a controlled-variable strategy by fixing the semi-major axis while systematically decreasing the semi-minor axis to obtain aspect ratios of 1:1, 2:1, 4:1, and 8:1, thereby independently separating the total vacancy area from the localized crack-tip curvature.

Furthermore, investigating elliptical defects in ψ-graphene is fundamentally distinct from a routine parameter extension of isotropic void models. Owing to the unique non-hexagonal 5–6–7 ring topology and orthogonal lattice symmetry of ψ-graphene, its localized deformation physics is intrinsically anisotropic. While symmetric circular voids impose predominantly isotropic stress concentration, introducing elliptical nanoholes with controllable aspect ratios and tilt angles breaks local structural symmetry and induces a transition from pure tensile fracture to a mixed-mode (tensile and shear) failure mechanism. Such lattice-governed stress redistribution and crack-propagation trajectories cannot be captured by symmetric holes or isotropic honeycomb lattices. Therefore, systematic probing of these geometric parameters and their interplay with anisotropy is crucial for revealing bond-rupture energy barriers, guiding defect engineering, and enabling reliability assessment of ψ-graphene-based components.

In this work, we employ MD simulations to systematically investigate the mechanical properties and failure mechanisms of ψ-graphene containing elliptical nanoholes. We focus on the interplay between the defect aspect ratio, orientation, and loading direction under extreme conditions. Our results reveal a non-monotonic relationship between the aspect ratio and mechanical strength along different axes; for *AR* = 4 the mechanical performance reaches its minimum at a critical tilt angle of 75°. The remainder of this paper is organized as follows: Section 2 provides the computational details. Section 3 presents the simulation results and a comprehensive discussion of the findings. Finally, the conclusions of this work are summarized in the last section.

## 2. Materials and Methods

**Figure 1:**
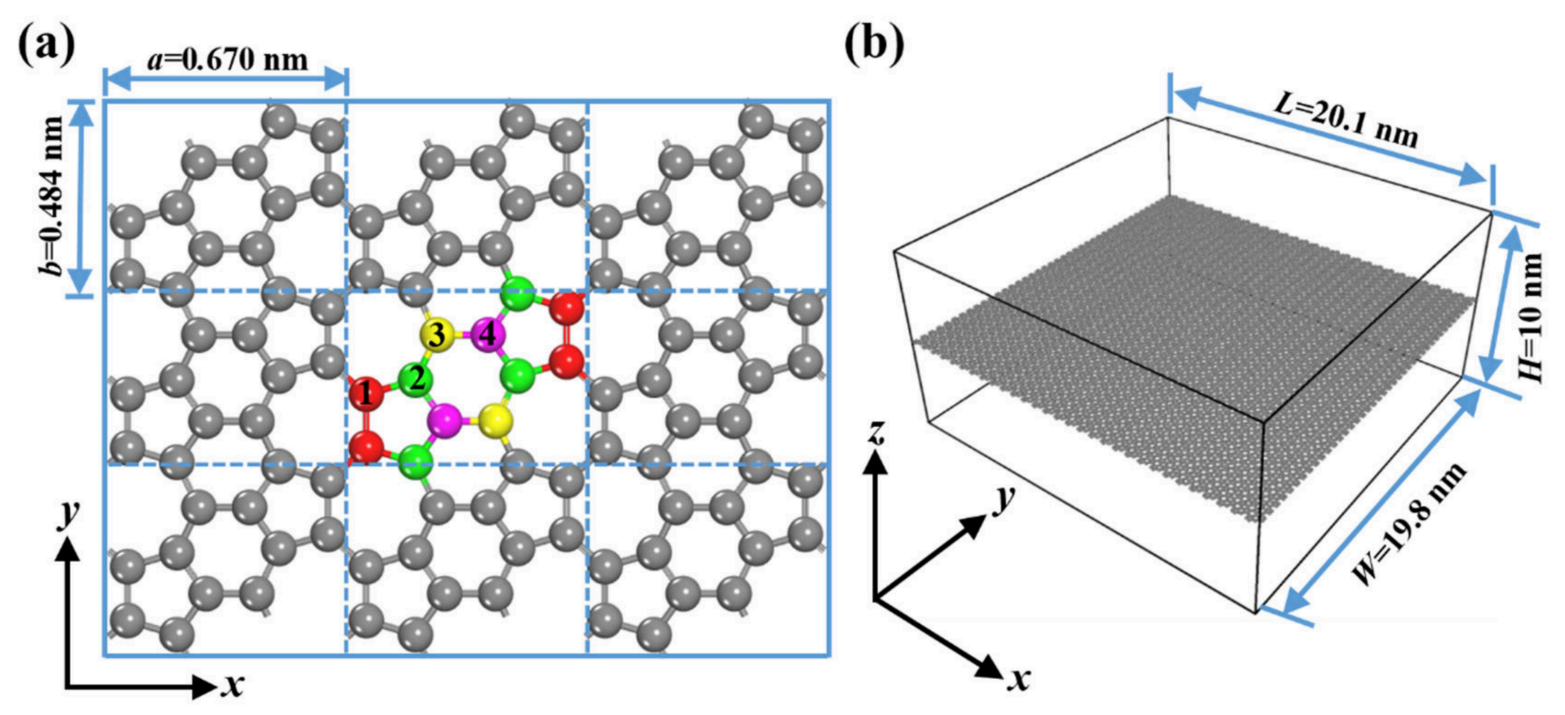
Atomic structure and computational model of ψ-graphene. (**a**) Monolayer ψ-graphene. Geometry of the unit cell with lattice parameters *a* = 0.670 nm and *b* = 0.484 nm. Non-equivalent carbon atoms are highlighted in different colors and labeled as 1–4. (**b**) The simulation setup for MD calculations. The simulation box dimensions are *L* = 20.1 nm, *W* = 19.8 nm, and *H* = 10 nm, with 14,760 carbon atoms.

The atomic configuration of ψ-graphene was first established and optimized. As illustrated in Figure 1a, ψ-graphene exhibits a planar structure with a single-atom thickness, analogous to graphene. The optimized lattice constants of the unit cell were determined to be *a* = 0.670 nm and *b* = 0.484 nm, which are in excellent agreement with previously reported results from first-principles calculations [9]. Within each unit cell, there are four symmetry-inequivalent carbon atoms, labeled as 1, 2, 3, and 4 in Figure 1a, which define the unique topology of the ψ-graphene lattice. The effective thickness value of ψ-graphene was set to 3.35 Å in accordance with the widely accepted effective thickness of graphene. For the MD simulations, a large-scale nanosheet model was constructed, as shown in Figure 1b. The dimensions of the simulation domain were set to approximately 20.1 nm × 19.8 nm along the *x* and *y* directions, respectively, to maintain a nearly square geometry and minimize the artifacts of finite-size effects. Periodic boundary conditions (PBCs) were applied in the *x*, *y* and *z* directions. The simulation box height was set to 10 nm, with the ψ-graphene sheet positioned in the center. This vacuum spacing is sufficient to prevent spurious interactions between the sheet and its periodic images in the *z*-direction. All atomic structural evolutions and defect configurations were visualized using the OVITO Basic (version 3.10.6) [23].

**Figure 2:**
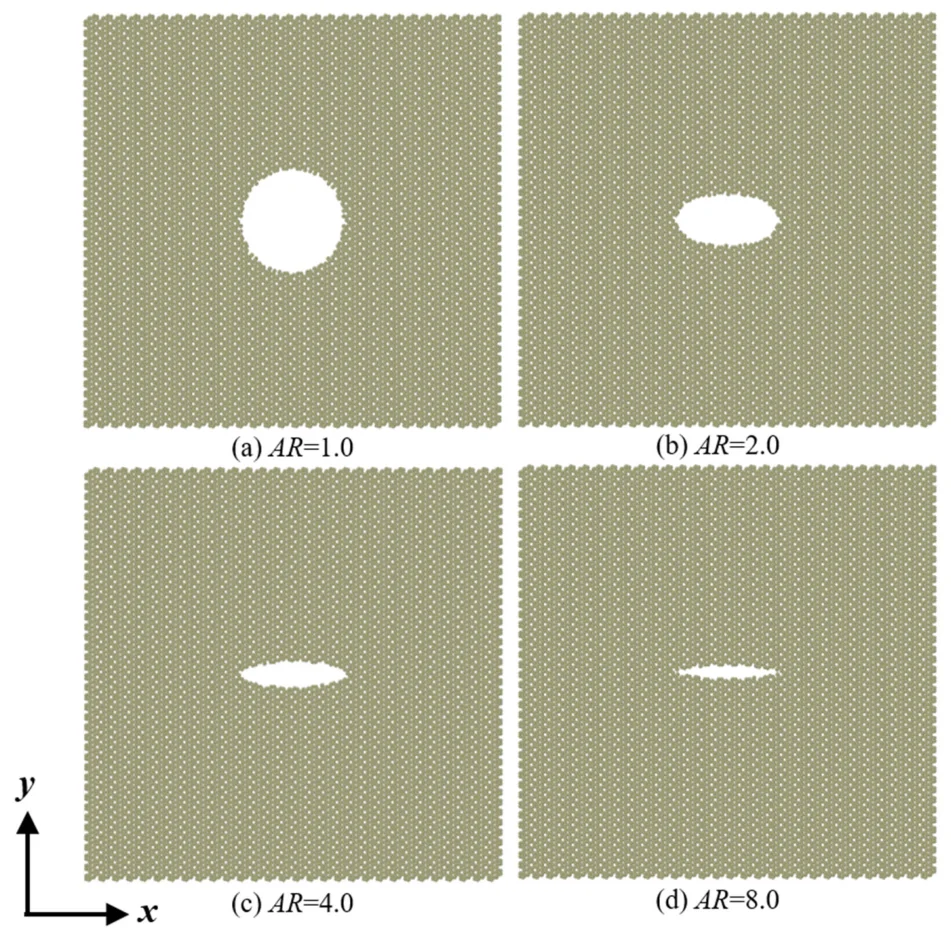
Geometric models of ψ-graphene with elliptical holes of varying aspect ratios (*AR*). The center of each sheet contains an elliptical hole with a fixed major axis length, while the aspect ratio is varied as: (**a**) *AR* = 1.0 (circular hole), (**b**) *AR* = 2.0, (**c**) *AR* = 4.0, and (**d**) *AR* = 8.0. The major axis of the elliptical holes is aligned parallel to the *x*-axis.

To investigate the influence of defect geometry on the mechanical properties of ψ-graphene, elliptical nanoholes with varying aspect ratios were introduced into the center of the pristine nanosheets of Figure 1b. As depicted in Figure 2, four representative models were constructed with different aspect ratios of 1, 2, 4, and 8, respectively, by keeping the semi-major axis constant while systematically varying the semi-minor axis. The overall dimensions of these defective nanosheets remain identical to the pristine structure described in Figure 1, ensuring that any observed variations in mechanical performance are solely attributable to the geometric characteristics of the elliptical defects.

The MD simulations were performed using the Large-scale Atomic/Molecular Massively Parallel Simulator (LAMMPS) package (27 June 2024) [24]. To describe the interatomic interactions between carbon atoms in ψ-graphene, the AIREBO-M potential was employed, which has been widely proven to accurately capture the mechanical properties and fracture behaviors of carbon-based 2D materials [25,26,27,28,29,30]. Before loading, the systems were fully relaxed to their minimum energy state using the conjugate gradient (CG) algorithm, followed by an equilibration process in the isothermal-isobaric (NPT) ensemble at a temperature of 300 K for 500 ps under zero pressure. Here the Nosé–Hoover thermostat and barostat were utilized for temperature and pressure regulation. The velocity-Verlet algorithm was employed to integrate the Newton equations with a time step of 1 fs.

**Figure 3:**
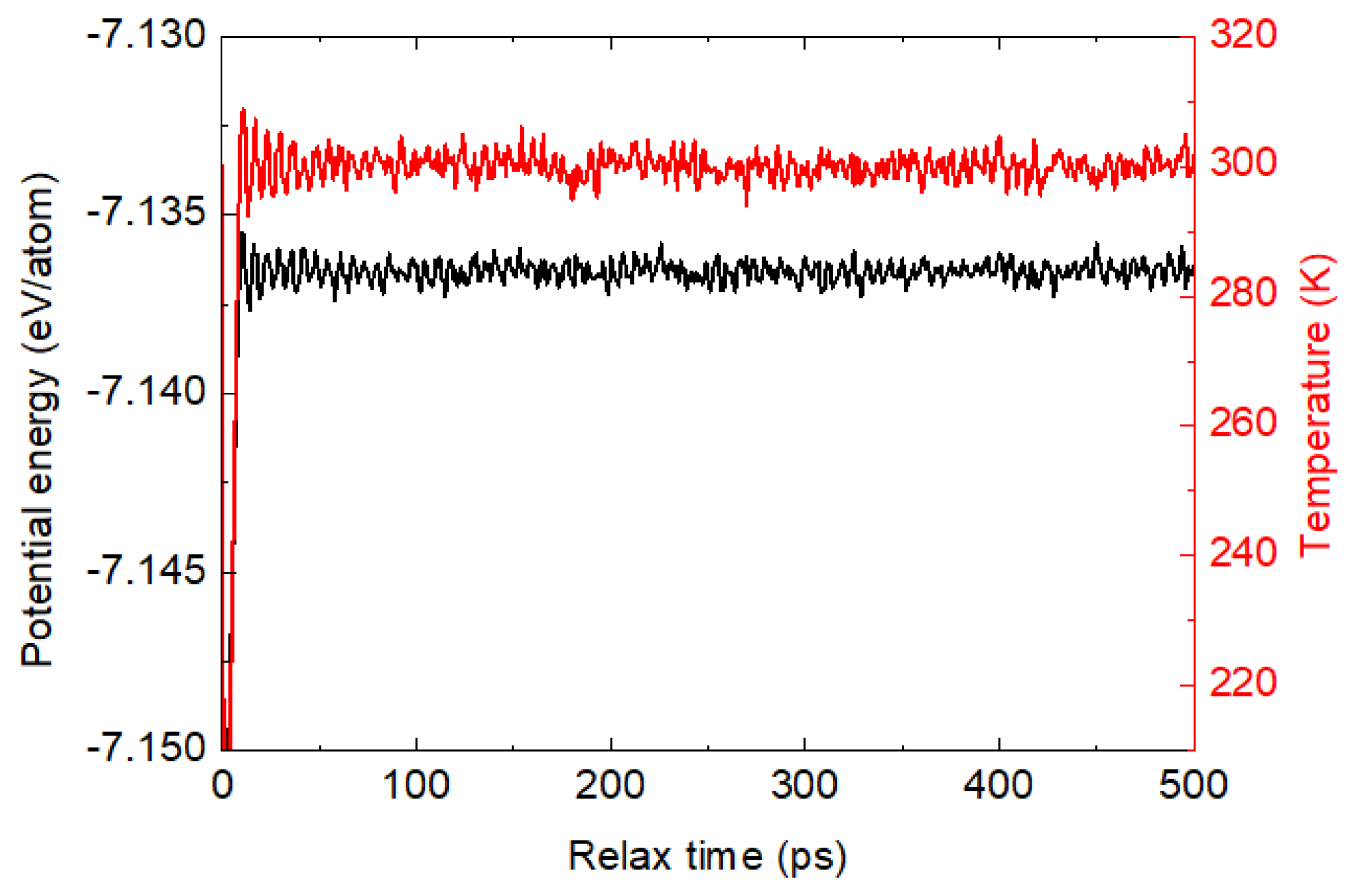
Structural relaxation of the ψ-graphene system prior to tensile loading. The evolution of the (left axis) potential energy per atom (black line) and (right axis) system temperature (red line) is shown as a function of the relaxation time (ps).

To verify system equilibration, the potential energy per atom and temperature of the pristine ψ-graphene nanosheet were monitored over a 500 ps period. As shown in Figure 3, both parameters converged rapidly within the initial tens of picoseconds and remained stable throughout the remainder of the simulation. This steady-state behavior confirms that 500 ps is sufficient to achieve complete thermodynamic equilibrium. Particularly, a rigorous box dimension constraint was implemented to prevent the introduction of artificial thermal stresses during the ensemble transition. During the 500 ps isothermal-isobaric (NPT) relaxation under zero pressure, the instantaneous dimensions of the simulation domain fluctuated naturally due to thermal vibrations. To eliminate these numerical artifacts, the equilibrium lattice parameters were determined by taking the time-average of the box dimensions over the final 200 ps of the NPT run. Prior to the onset of uniaxial tensile loading, the simulation box was fixed to these time-averaged equilibrium dimensions, and the system was subsequently switched to the canonical (NVT) ensemble. This transition methodology could guarantee a zero-stress initial state and excellent numerical stability throughout the mechanical deformation process. The subsequent tensile loading was performed under the NVT ensemble at 300 K using the Nosé–Hoover thermostat to effectively dissipate the thermal energy generated during structural deformation. Similarly, this identical equilibration and transition procedure was uniformly applied to all defective ψ-graphene configurations to ensure that any residual localized stresses induced by atom removal were thoroughly dissipated. It should be noted that the strain rate is a key factor influencing the mechanical response of nanomaterials under tensile loading. In this study, considering both the computational efficiency and the requirement for numerical stability, a constant strain rate of 10^8^ s^−1^ was applied along the loading direction. This value was chosen in accordance with previous literature on 2D carbon allotropes to ensure that the structural evolution and fracture mechanisms are captured with sufficient accuracy while remaining within a manageable computational timeframe [31,32,33].

In order to quantify the mechanical response, strain and stress values were systematically captured using LAMMPS. Specifically, the uniaxial strain (*ε*) is defined as $\varepsilon \;=\;\left(L-L_{0}\right)/L_{0}$, where *L*_0_ represents the initial equilibrium length of the simulation box along the loading direction, and *L* represents the instantaneous length under applied strain. At the macroscopic scale, the tensile stresses were reported as force per unit width (N/m), which was free from any arbitrary physical thickness assumptions. Tensile stresses provide a representative two-dimensional measure of the mechanical strength. At the microscopic scale, the scalar equivalent atomic von Mises stress was evaluated to analyze localized stress concentrations and structural evolution during the tensile process. The atomic von Mises stress was expressed in GPa by assuming an effective monolayer thickness of h = 3.35 Å. After the axial tension simulations, the stress–strain profiles were analyzed to determine the fracture strain, fracture stress, Young’s modulus (*Y_s_*), and toughness. The fracture strain and stress represent the maximum deformation and load-bearing capacity of the ψ-graphene sheet, respectively. Young’s modulus, which characterizes the elastic stiffness, was derived from the slope of the linear-elastic regime within the small-strain interval of 0–0.015. Additionally, the toughness, representing the energy absorption capacity per unit area, was quantified by integrating the area under the stress–strain curve from zero strain to the fracture point.

To ensure the statistical reliability of these extracted mechanical parameters against stochastic thermal fluctuations, independent benchmark simulations under *x*-axis loading were subsequently performed using four distinct random velocity seeds for both the pristine system and the defective structures across different aspect ratios (*AR* = 1, 2, 4, and 8). The key mechanical parameters extracted from these independent runs are thus reported as mean ± standard deviation (SD) to ensure statistical validity.

## 3. Results

### 3.1. Model Validation and Mechanical Properties of Pristine ψ-Graphene

To validate the reliability of the adopted classical molecular dynamics model and the calibrated AIREBO-M potential parameters, as well as to establish a reference baseline for discussing the mechanical properties of defective structures, we first focused on the mechanical performance of pristine ψ-graphene at 300 K. Specifically, its Young’s moduli along the *x*- and *y*-axes were calculated. The calculated results are summarized and compared with established literature values obtained from density functional theory (DFT) calculations and independent MD simulations in Table 1. Our simulations yield a Young’s modulus of 266.88 N/m along the *x*-axis and 288.95 N/m along the *y*-axis, which demonstrates acceptable consistency with the 0 K DFT data reported by Ref. [9] ($Y_{s}^{x}$ = 283.49 N/m, $Y_{x}^{y}$ = 298.17 N/m) and another independent DFT study ($Y_{s}^{x}$ = 272.58 N/m, $Y_{s}^{y}$ = 280.70 N/m) [11]. The discrepancy between our values and the DFT predictions is primarily attributed to the effect of thermal fluctuations. At 300 K, the active kinetic energy of carbon atoms induces intrinsic out-of-plane wrinkles and thermal vibrations, which naturally alleviate the in-plane structural resistance and result in a slightly lower stiffness compared to absolute zero. Furthermore, our values are good agreement with the independent MD results from Ref. [12] ($Y_{s}^{x}$ = 264.38 N/m, $Y_{s}^{y}$ = 275.04 N/m) using the identical AIREBO-M potential at 300 K. The slight discrepancy may primarily arise from their use of the NEMD method and a different ensemble. Therefore, this high level of consistency ensures the reliability of our computational framework, allowing us to extend our investigation to the mechanical response of ψ-graphene containing various elliptical nanoholes.

### 3.2. Effect of Aspect Ratio Under Principal Axis Loading

**Figure 4:**
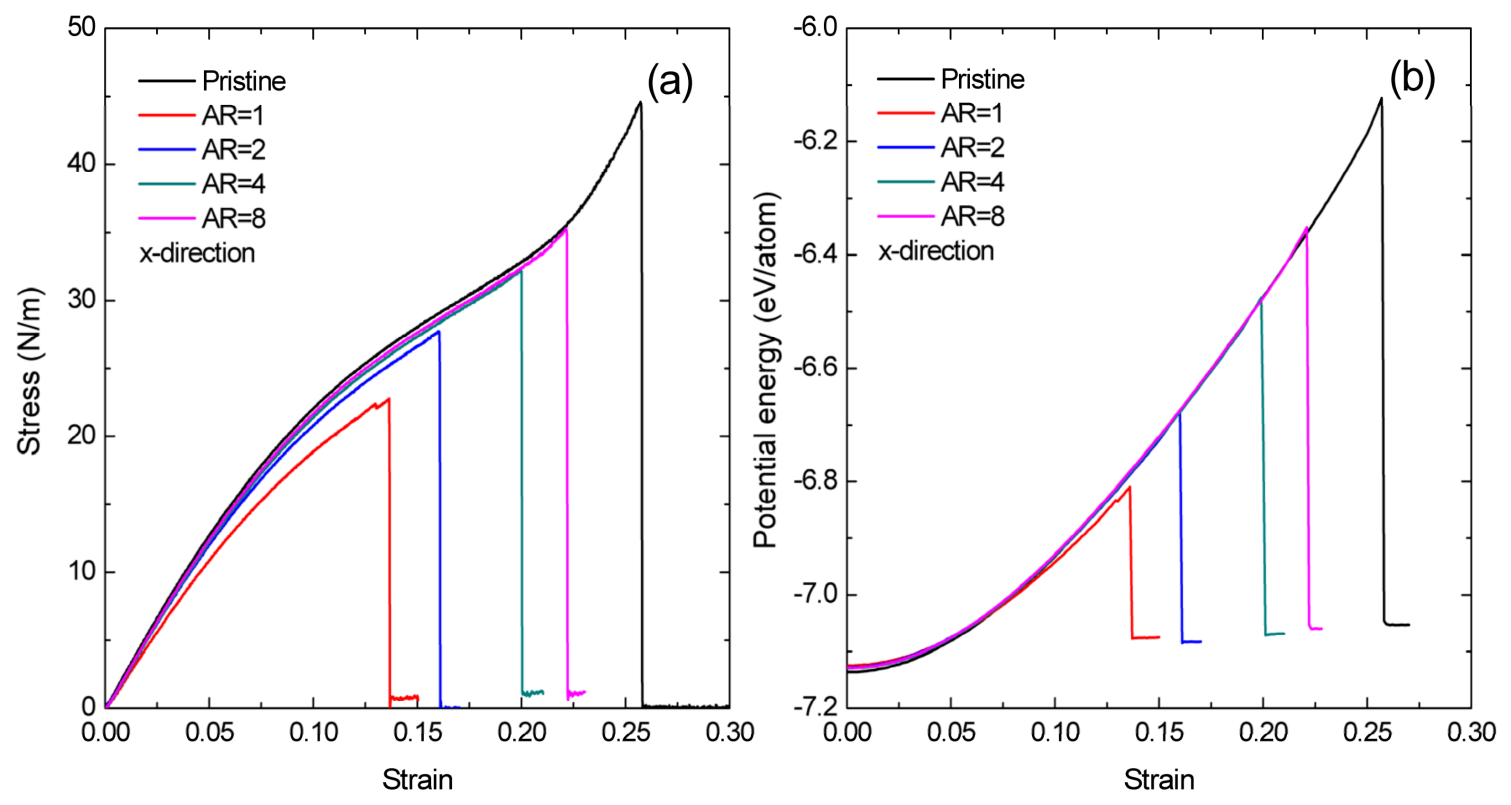
Representative mechanical response of ψ-graphene with elliptical defects of various aspect ratios under tensile loading along the *x*-direction. (**a**) Stress–strain curves; (**b**) evolution of average potential energy per atom as a function of strain. The results for pristine ψ-graphene are also provided for comparison.

As described in the Computational Details section, independent simulations using four distinct random velocity seeds were performed for both the pristine and defective systems (*AR* = 1, 2, 4, and 8) under *x*-axis loading. Since the stress–strain evolution with different random seeds is qualitatively identical with negligible numerical variations, only the curves from a single representative seed are presented and discussed here, as shown in Figure 4a. Through these specific elliptical defect geometries, we evaluate the influence of aspect ratio on the mechanical integrity of the material. All curves exhibit a linear elastic regime at low strain levels, followed by a nonlinear increase until a sudden drop in stress occurs, signifying the brittle fracture of the ψ-graphene nanosheets. The pristine ψ-graphene possesses the highest fracture stress of 44.45 N/m and fracture strain of 0.2598. The introduction of elliptical nanoholes leads to a significant degradation in both strength and ductility, which is attributed to the stress concentration near the defect edges. A compelling observation from Figure 4 is the non-monotonic dependence of the mechanical properties on the aspect ratio when stretching along the *x*-direction. For the circular defect (*AR* = 1), the stress–strain curve shows the lowest tensile strength (~23 N/m) and fracture strain (~0.14). In this case, the circular hole creates a substantial transverse discontinuity relative to the loading direction, leading to severe stress localization. Interestingly, as *AR* increases from 1 to 8, both the fracture stress and fracture strain exhibit a progressive recovery. This phenomenon can be explained by two aspects as follows. First, when the major axis of the elliptical defect is parallel to the loading direction, the defect becomes more streamlined, the curvature radius at the longitudinal tips increases, effectively reducing the local stress gradient compared to a circular hole. Second, an ellipse with a higher *AR* occupies a narrow span in the y-direction. Consequently, a larger portion of the ψ-graphene lattice remains intact to bear the applied load, leading to the enhanced load-carrying capacity. The curves also show that the Young’s modulus (the initial slope) remains relatively consistent across all defected samples, suggesting that the global elastic stiffness is less sensitive to the specific *AR* than the ultimate strength.

To ensure the physical validity of these observed trends, it should be noted that the choice of the potential cutoff radius (*r*_cutoff_) has a negligible impact on our core conclusions. As demonstrated in Figure S1 for loading along the *x*-axis, evaluating different values of *r*_cutoff_ (2.0, 2.1, 2.2, and 2.25 Å) reveals that the stress–strain curves of the pristine ψ-graphene almost perfectly overlap in the strain region below 0.22 (Figure S1a). More importantly, for the defective sheets (*AR* = 4), the four stress–strain curves perfectly coincide throughout the entire loading process until abrupt failure (Figure S1b). Since crack propagation occurs prematurely at moderate strains (*ε* ≈ 0.19–0.21), most C-C covalent bonds do not reach the critical extension thresholds that activate the AIREBO-M potential cutoff stiffening anomaly. Consequently, the fracture behavior is predominantly governed by the localized stress concentration at the defect tips, rather than the potential cutoff distance.

Beyond the potential cutoff parameters, the potential influence of finite-size effects and periodic boundary condition artifacts was systematically evaluated. To this end, size-convergence tests were performed by doubling the simulation domain to a larger system of 40.2 nm × 39.6 nm. The absolute physical dimensions of the nanoholes were held strictly constant to isolate boundary effects from geometric scaling. As illustrated in Figure S2, two representative cases were investigated: the circular nanohole (*AR* = 1) with the maximum void area fraction, and the highly elongated elliptical nanohole (*AR* = 4) inducing severe local stress concentration. For both cases, the stress–strain profiles, Young’s moduli, and failure modes computed from the 40 nm systems exhibit remarkable alignment with the original 20.1 nm × 19.8 nm models. The minor variance in fracture stress (about 5.1% for *AR* = 1 and <1.0% for *AR* = 4) convincingly demonstrates that a simulation domain of 20.1 nm × 19.8 nm is sufficiently converged to eliminate periodic image interactions. Consequently, this configuration was adopted as the standard domain size throughout all main simulations to balance computational economy with physical accuracy.

To further elucidate the failure mechanism from a thermodynamic perspective, the evolution of the average potential energy per atom as a function of strain is presented in Figure 4b. The energetic response is found to be highly consistent with the stress–strain behavior shown in Figure 4a. During the initial elastic deformation stage, the potential energy increases quadratically, signifying the continuous accumulation of strain energy within the atomic bonds of the ψ-graphene lattice. Upon reaching the critical strain, a sharp drop in potential energy is observed, which serves as a clear indicator of structural rupture and the rapid release of stored elastic energy. Notably, the maximum energy-bearing capacity before fracture follows the same trend as the tensile strength regarding the different aspect ratios. The sample with a circular defect reaches the lowest peak potential energy, whereas increasing the *AR* along the *x*-direction allows the system to sustain higher energy states before failure. This high accordance between stress and energy evolution reinforces our conclusion that aligning the major axis of the elliptical defect with the loading direction effectively mitigates strain energy localization, thereby delaying the onset of bond dissociation and enhancing the overall mechanical stability.

**Figure 5:**
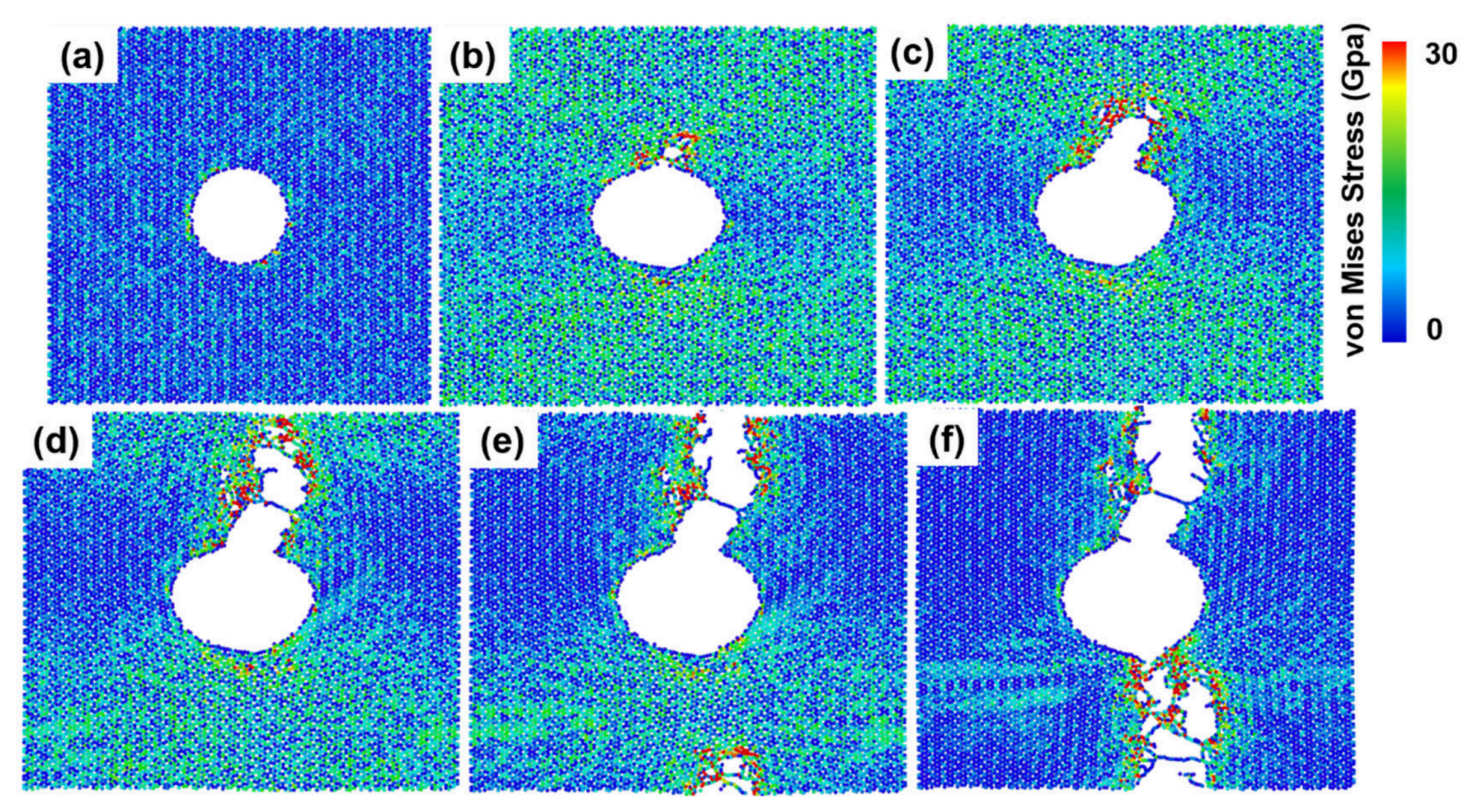
Evolution of crack formation and propagation for a circular defect (*AR* = 1) in ψ-graphene and the corresponding von Mises stress distribution under *x*-axis tension. The snapshots (**a**–**f**) illustrate the progressive stages of structural failure under loading. The color contour represents the von Mises stress field, ranging from 0 GPa (blue) to 30 GPa (red). High stress concentration is initially observed at the hole boundaries, leading to crack initiation and subsequent propagation until global structural failure occurs in stage (**f**).

Figure 5a–f provides a microscopic perspective on the structural degradation by illustrating the evolution of von Mises stress distribution for the sample with a circular defect (*AR* = 1). As shown in Figure 5a, the initial snapshot represents the equilibrated state after structural optimization but prior to the application of tensile loading, characterized by a uniform and minimal stress distribution across the ψ-graphene lattice. Upon the commencement of uniaxial tension, pronounced stress concentration emerges at the transverse poles of the nanohole. As the strain approaches the critical fracture point (Figure 5b,c), these high-stress “hotspots” trigger the initial bond dissociation, leading to crack initiation at the defect boundary. Subsequently, the crack propagates rapidly along the direction perpendicular to the loading axis (Figure 5d,e). The final catastrophic rupture (Figure 5f), marked by the complete loss of cross-sectional integrity, perfectly mirrors the abrupt stress drop observed in Figure 4a, confirming that the defect-induced stress localization is the primary origin of the premature failure in ψ-graphene.

**Figure 6:**
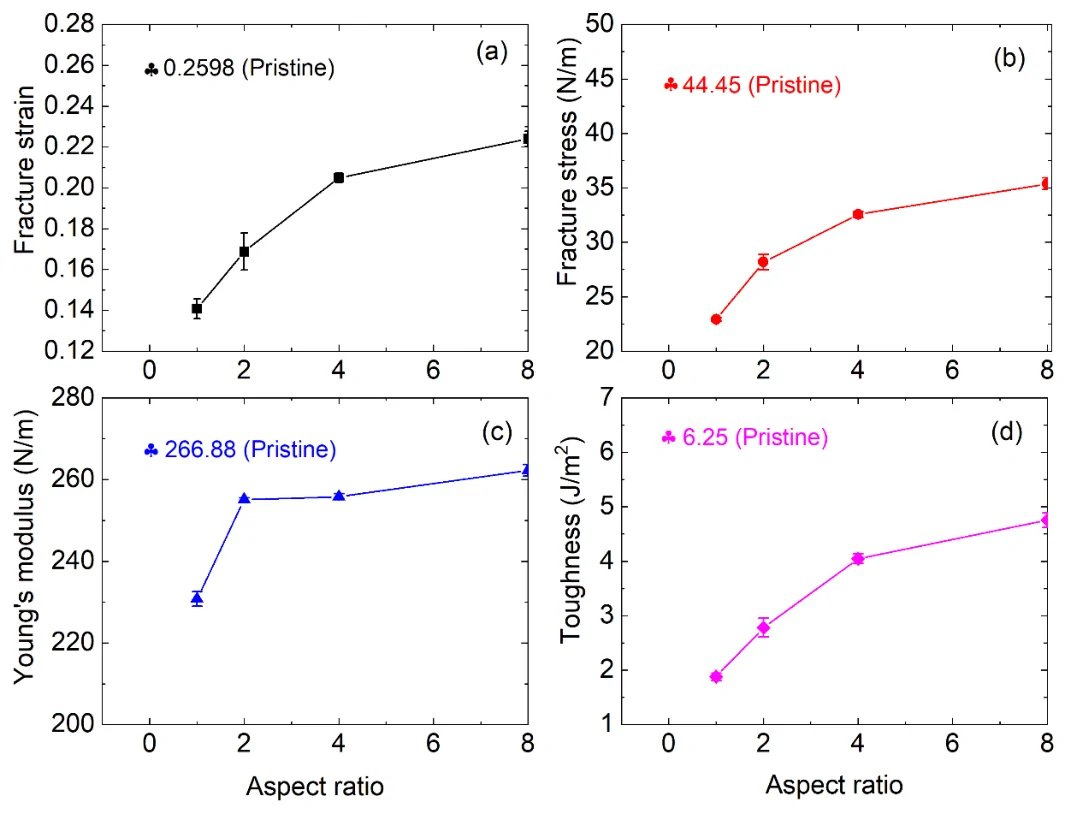
Mechanical properties of defective ψ-graphene under *x*-axis loading as a function of aspect ratio (*AR*). (**a**) Fracture strain, (**b**) Fracture stress, (**c**) Young’s modulus, and (**d**) Toughness as a function of aspect ratio (*AR*). Data points and error bars represent the mean values and corresponding standard deviations (SD) obtained from four independent simulations with distinct random velocity seeds. The plum blossom denotes the reference value for the Pristine state.

To systematically evaluate the impact of defect geometry, the fracture strain, fracture stress, Young’s modulus, and toughness under x-axis loading are extracted and plotted as a function of the aspect ratio in Figure 6a–d. As depicted in these profiles, the summarized mechanical parameters are plotted with error bars quantified from four independent statistical runs. The remarkably small standard deviations across all aspect ratios confirm that the observed variations in these mechanical properties are deterministically governed by geometric lattice-defect interactions rather than stochastic thermal fluctuations. The corresponding properties of pristine ψ-graphene are indicated by the plum blossom symbols as reference benchmarks. The introduction of a circular defect causes a severe degradation in mechanical performance. Compared to the pristine ψ-graphene (0.2598 and 44.45 N/m), the fracture strain and stress for ψ-graphene with a circular defect drop to 0.1408 and 22.92 N/m, representing a reduction of 45.8% and 48.4%, respectively. As the *AR* increases to 8, these values recover to 0.224 and 35.38 N/m. This recovery indicates that elongating the defect along the loading direction significantly restores the material’s load-bearing capacity, although it remains below the pristine level. The Young’s modulus exhibits a unique sensitivity trend. While the *AR* = 1 sample shows a noticeable decrease (230.74 N/m) from the pristine value (266.88 N/m), the values for *AR* = 2 to *AR* = 8 show a remarkable plateau, fluctuating only slightly between 255.10 and 262.24 N/m. This negligible variation (less than 3%) suggests that once the initial structural continuity is broken by a defect of a certain area, the elastic stiffness becomes relatively insensitive to the specific aspect ratio. In the linear-elastic regime, the global stiffness is primarily governed by the total effective cross-sectional area of the atomic bonds, which remains nearly constant across the different elliptical configurations. The toughness, quantifying the energy absorption until rupture, shows the most significant recovery. From *AR* = 1 (1.88 J/m^2^) to *AR* = 8 (4.76 J/m^2^), the toughness increases by approximately 153%. This substantial enhancement is a combined result of the recovered fracture stress and the significantly extended strain range, demonstrating that a higher *AR* effectively delays catastrophic failure and allows the ψ-graphene sheet to dissipate more strain energy.

**Figure 7:**
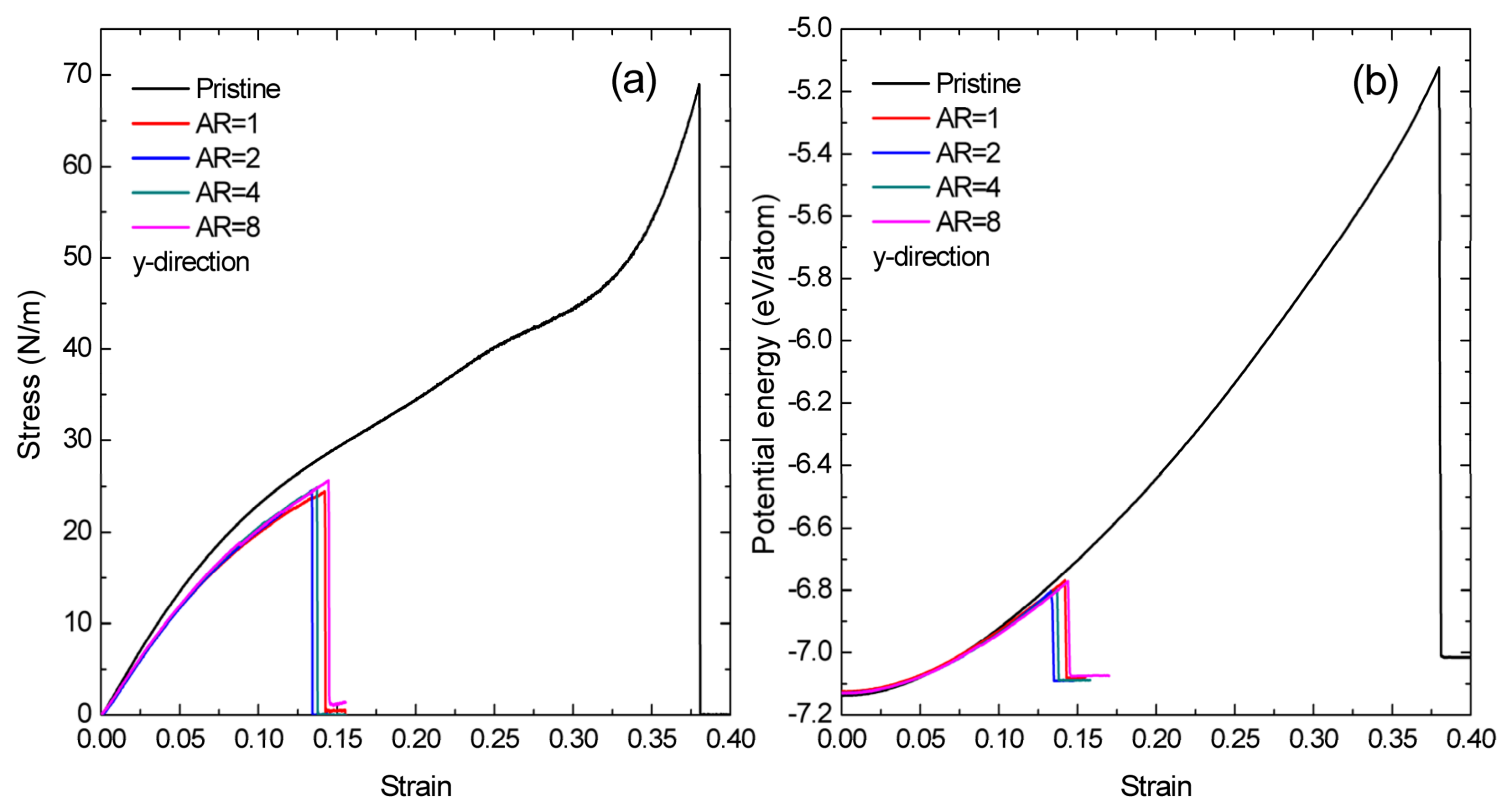
Mechanical response of ψ-graphene with elliptical defects of various aspect ratios under tensile loading along the y-direction. (**a**) Stress–strain curves; (**b**) evolution of average potential energy per atom as a function of strain. The results for pristine ψ-graphene are also provided for comparison.

The mechanical behavior of ψ-graphene under uniaxial tension along the *y*-direction is presented in Figure 7a. Similar to the *x*-direction results, all samples exhibit a linear elastic response followed by brittle fracture. However, a stark contrast is observed regarding the influence of the defect’s aspect ratio. While the pristine ψ-graphene shows a remarkably high tensile strength of approximately 68.94 N/m and a fracture strain of 0.3804, the introduction of elliptical defects leads to a catastrophic decline in performance. Interestingly, in the *y*-loading case, increasing the *AR* from 1 to 8 does not result in the blunting effect observed previously. Instead, all defected samples cluster at a much lower strength level (around 24–26 N/m). This is because the major axis of the elliptical defect is oriented perpendicular to the loading direction (*y*-axial). This configuration maximizes the crack-like behavior of the defect, where the sharp tips of the ellipse act as critical points for stress concentration, leading to premature failure at significantly lower strain levels (~0.14) regardless of the *AR* value.

The corresponding average potential energy per atom for *y*-direction tension is shown in Figure 7b, providing further thermodynamic evidence of the material’s failure. The potential energy curves for all defected samples nearly overlap, reflecting the identical trend observed in their stress–strain profiles in Figure 7a. Compared to the pristine sheet, which can reach a high energy state of −5.1 eV/atom before rupture, the defected samples fail at a much lower energy limit of approximately −6.8 eV/atom. This unified energy drop-off suggests that when the major axis of an ellipse is perpendicular to the load, the structural instability is triggered almost immediately upon reaching a critical local bond energy at the sharp elliptical tips. This pronounced discrepancy between the *x*- and *y*-axial mechanical responses indicates the strong anisotropy of defected ψ-graphene. This direction-dependent behavior stems from the relative orientation between the defect’s major axis and the loading direction. Under *x*-loading, increasing the *AR* induces a geometric blunting effect that alleviates stress concentration, whereas under *y*-loading, the defect acts as a transverse crack with sharp tips that maximize stress localization regardless of the *AR*. Furthermore, the intrinsic mechanical anisotropy of ψ-graphene plays a crucial role, as the material is inherently stronger but more strain-sensitive along the *y*-axis. This property amplifies the disparity and leads to the observed divergence in defect tolerance between the two principal directions.

**Figure 8:**
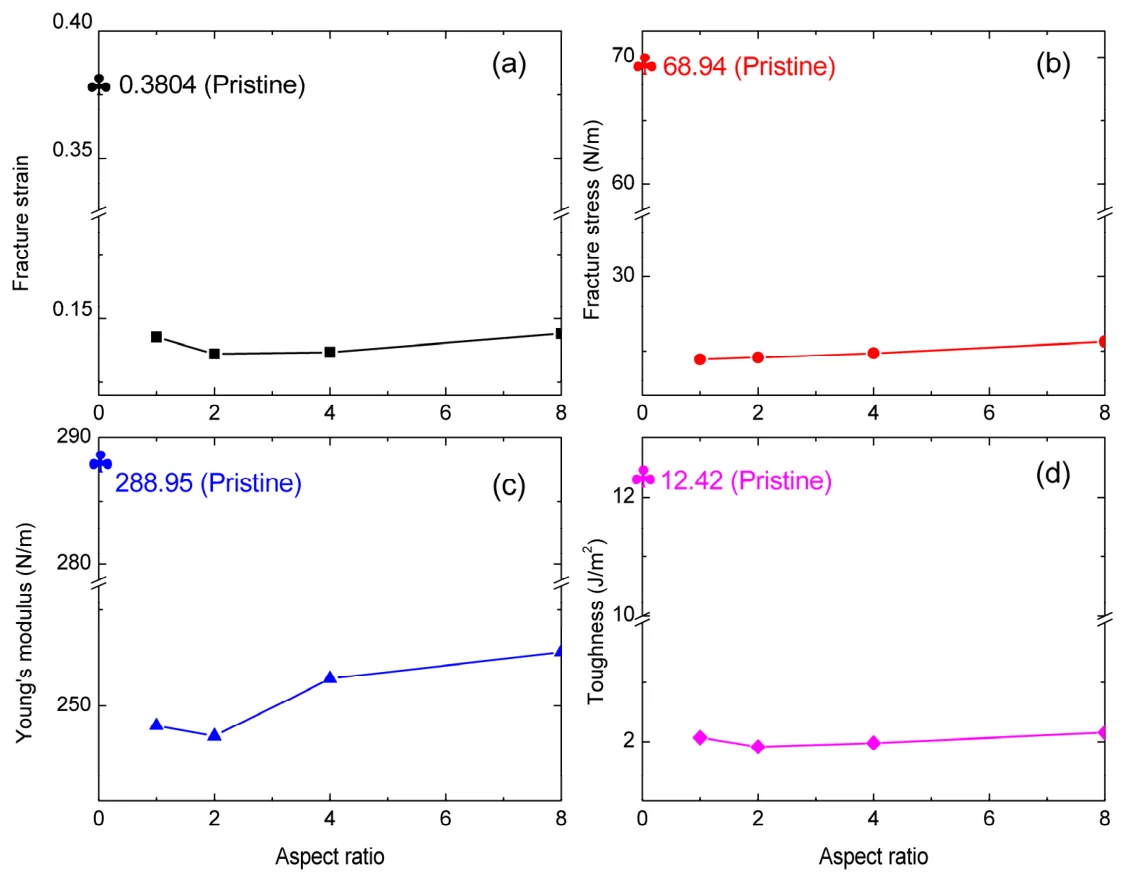
Comparative analysis of mechanical properties between pristine ψ-graphene and models with elliptical holes (*AR* = 1–8). To visualize the significant performance gap, *y*-axis breaks are employed to accommodate the high values of the pristine state (marked by plum blossoms) alongside the recovery trends of the defective sheets. (**a**) Fracture strain, (**b**) fracture stress, (**c**) Young’s modulus, and (**d**) toughness.

To quantify the observed anisotropic response, the key mechanical parameters under *y*-direction tension are extracted and presented as a function of the aspect ratio in Figure 8. Unlike the behavior observed under *x*-loading, the mechanical performance along the *y*-axis exhibits a persistent degradation that is largely independent of the defect’s elongation. The introduction of a circular defect leads to a precipitous drop in both fracture strain and tensile strength, declining from the pristine values (0.3804 and 68.94 N/m) to 0.143 and 24.44 N/m, respectively. As the *AR* increases from 1 to 8, these values remain remarkably stable, showing only negligible variations. This trend confirms that when the defect’s major axis is perpendicular to the loading direction, the sharpening of the elliptical tips maintains a high level of stress concentration, preventing the structural recovery observed in the *x*-direction. Similarly, the Young’s modulus for the defected samples remains significantly lower than that of the pristine sheet (288.95 N/m). While there is a slight upward trend as the *AR* increases, the overall values (ranging from 248.94 to 252.81 N/m) represent a substantial loss in stiffness compared to the perfect lattice. This suggests that transverse elliptical defects impose a more severe disruption to the atomic bond-stretching network along the *y*-axis than longitudinal ones. The toughness highlights the most dramatic impact of orientation-dependent failure. The energy absorption capacity collapses from 12.42 J/m^2^ for the pristine sample to approximately 2 J/m^2^ for all defected configurations. This nearly 84% reduction across all *AR* values highlights the extreme sensitivity of ψ-graphene to transverse geometric nanoholes, where the material’s ability to dissipate strain energy is critically suppressed by the rapid crack-like initiation at the elliptical tips.

### 3.3. Defect Orientation Effects and Microscopic Failure Mechanisms

**Figure 9:**
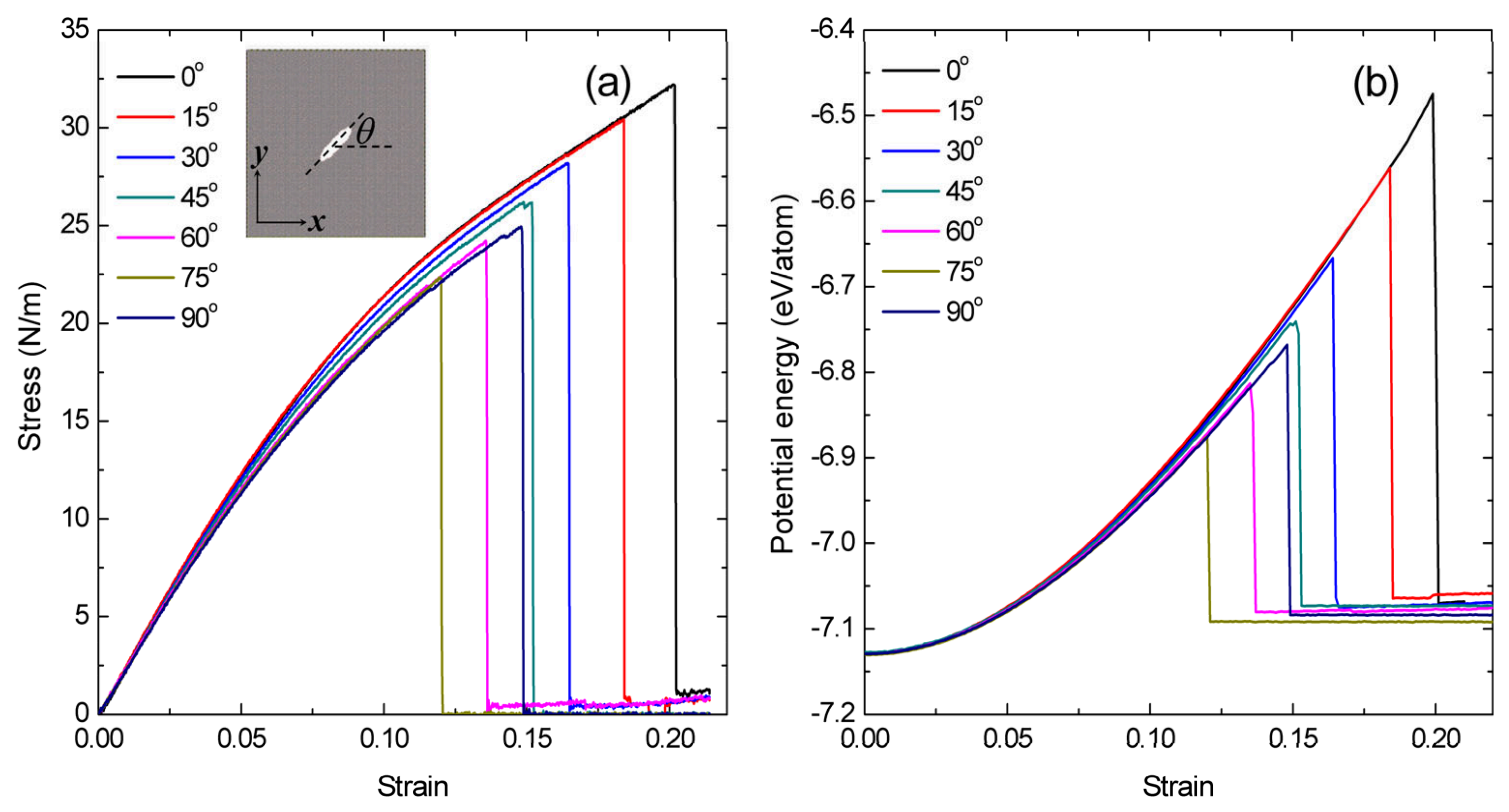
Geometric model and deformation process. (**a**) Tensile stress–strain curves and (**b**) evolution of potential energy per atom as a function of strain for ψ-graphene with an elliptical hole at various tilt angles (*θ*). The inset in (**a**) shows the schematic of the simulation model and the definition of the tilt angle *θ* relative to the loading direction.

Having established the impact of defect geometry along the principal axes, we now investigate the role of the defect’s orientation relative to the loading direction (*x*-direction). Specifically, the elliptical defect with a fixed aspect ratio (*AR* = 4) is rotated from *θ* = 0° to 90°, as illustrated in the inset of Figure 9a. The stress–strain behavior shown in Figure 9a is highly sensitive to variations in the defect’s inclination angle. As the angle *θ* increases from 0° to 90°, both the fracture stress and fracture strain exhibit a non-monotonic decline, reaching their minimum values at approximately *θ* = 75° rather than 90°. Specifically, the sample with *θ* = 0° (where the defect is aligned with the load direction) maintains the highest load-bearing capacity, while rotating the defect toward a vertical orientation significantly weakens the material. This trend suggests that the interaction between the tilted defect and the lattice’s intrinsic bonding network creates more complex stress concentration patterns than those observed in the simple aligned cases.

The average potential energy per atom, as shown in Figure 9b, mirrors the structural degradation as observed in the stress–strain profiles. Samples with smaller rotation angles (e.g., 0° and 15°) can sustain higher energy states before failure, indicating a greater capacity for elastic energy storage. In contrast, for larger angles, the potential energy drops abruptly at much lower strain levels. That is to say, energy curves for high-angle configurations ($\theta \geq 45^{{}^{\circ}}$) are significantly compressed. This confirms that tilted defects promote earlier bond rupture, as the sharp elliptical tips align with favorable fracture paths in the ψ-graphene lattice.

**Figure 10:**
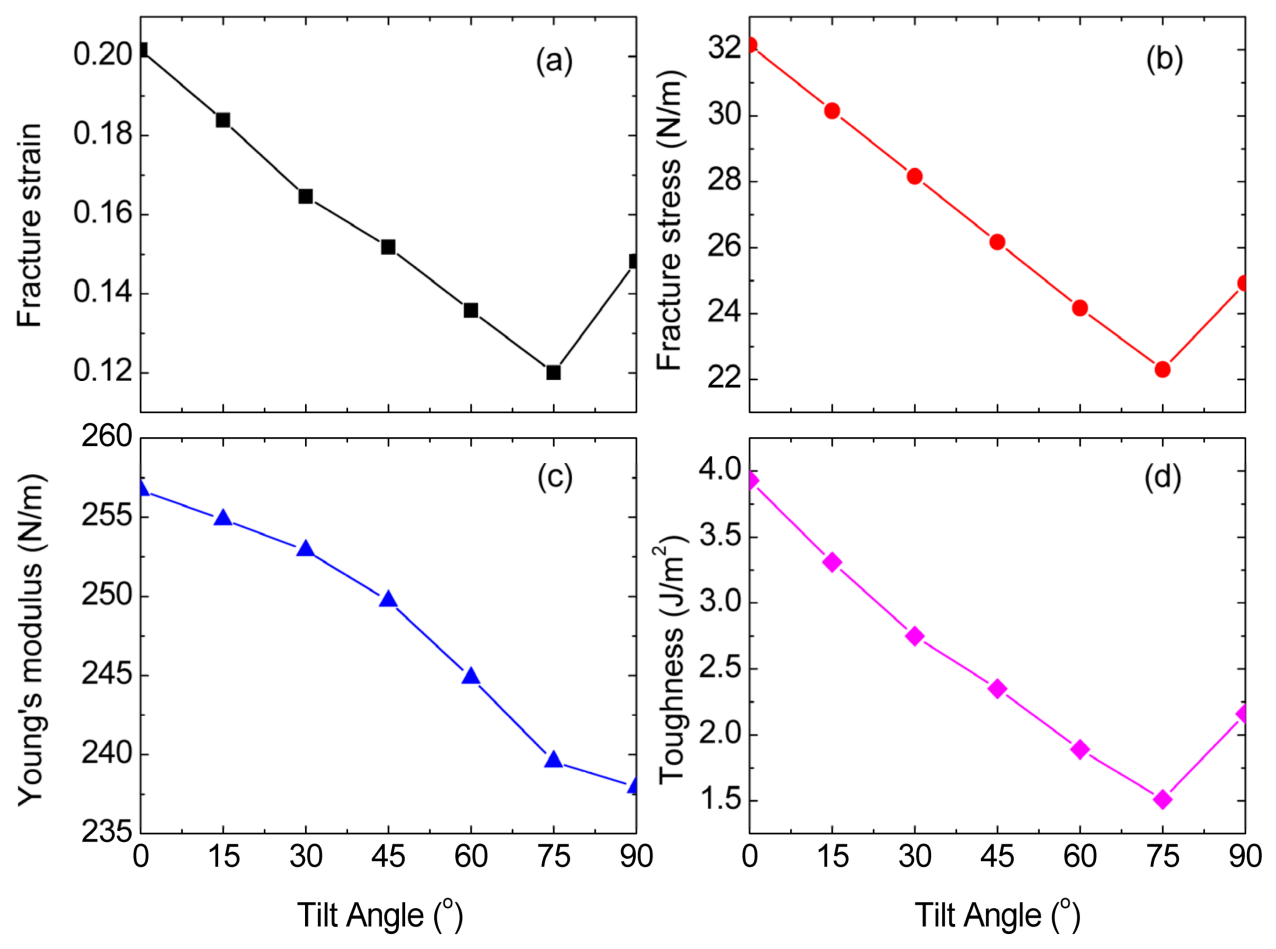
Mechanical properties of ψ-graphene with an elliptical defect as a function of the tilt angle under tensile loading along the *x*-axis. (**a**) Fracture strain, (**b**) fracture stress, (**c**) Young’s modulus, and (**d**) toughness versus the tilt angle of the defect. All mechanical indicators exhibit a consistent V-shaped trend, reaching their minimum values near *θ* = $75^{{}^{\circ}}$.

To provide a more detailed understanding of the orientation effect, the fracture strain, fracture stress, Young’s modulus, and toughness were calculated from the stress–strain curves and plotted as a function of the tilted angle (*θ*) in Figure 10a–d. Interestingly, the fracture strain, fracture stress, and toughness all exhibit a distinct non-monotonic decreasing trend as the tilt angle increases from 0° to 90°. The mechanical property does not reach its minimum at the tilted angle of 90°, but rather at a critical angle of 75°. For instance, the fracture stress drops from 32.1 N/m at 0° to a minimum of 22.3 N/m at 75°. However, the value slightly recovers to 24.9 N/m at 90°. We may explain this behavior as follows. The minimum performance at 75° is due to the defect aligning with lattice failure planes. Stress concentration is consequently maximized, facilitating rapid bond rupture. Unlike the other parameters, the Young’s modulus shows a continuous and monotonic decrease as the defect rotates from 0° to 90°. The modulus falls from 256.7 N/m to 237.9 N/m. This trend suggests that as the defect’s projection area perpendicular to the loading direction increases with *θ*, the effective cross-sectional stiffness of the sheet is steadily compromised. The mismatch between the monotonic decline of *Y_s_* and the non-monotonic trend of strength suggests that elastic stiffness is governed by global structural continuity, while fracture is triggered by local atomic instabilities. These quantitative findings are in excellent agreement with the energy profiles in Figure 9b. The sharpest drops in potential energy and the lowest energy-bearing capacities correspond precisely to the configurations near *θ* = 75°. The trend of toughness in Figure 10d closely mirrors that of fracture strain. This correlation confirms that energy dissipation is primarily limited by premature crack initiation at these critical angles.

**Figure 11:**
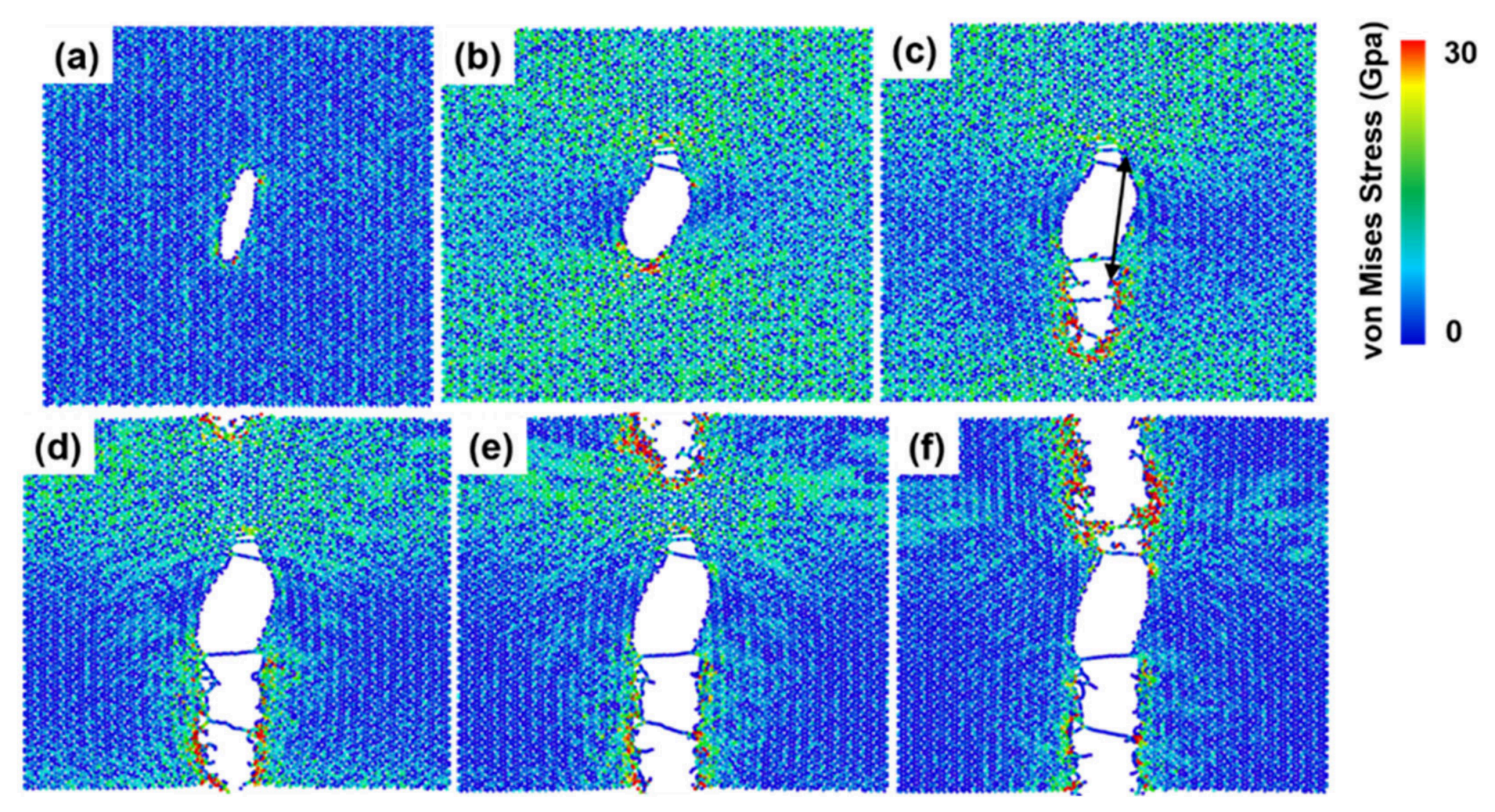
Evolution of crack propagation and corresponding stress distribution in ψ-graphene with a 75° inclined elliptical defect under x-axis tension. (**a**–**f**) Sequential stages of material failure under loading. The color contours represent the von Mises stress distribution (in GPa), highlighting the stress concentration at the crack tips. The black arrow in (**c**) indicates the primary loading direction/crack opening mode.

To further investigate the underlying mechanism responsible for the minimum mechanical performance observed at *θ* = 75°, the evolution of the von Mises stress distribution is illustrated in Figure 11a–f. In the relaxed state prior to loading, just as shown in Figure 11a, the stress field around the elliptical nanohole is uniform and minimal, indicating that the defect is well-accommodated within the ψ-graphene lattice without spontaneous structural distortion. As the tensile strain approaches the failure threshold, high-stress regions (indicated by the red-colored atoms) rapidly emerge at the sharp tips of the tilted ellipse. Notably, Figure 11c shows that the stress concentration is not symmetrically distributed but follows the tilted orientation of the defect. This creates a specific stress pathway that orients along the weaker bonding directions of the lattice, eventually triggering the localized rupture of atomic bonds at the elliptical tips. Once initiation occurs, the stress is redistributed toward the newly formed crack surfaces. The micro-cracks propagate rapidly along a path nearly perpendicular to the loading direction, as seen in the transition from Figure 11d–f. For this elliptical defect with a 75° tilted angle, the hexagonal and pentagonal rings break with minimal energy. This explains the sharp decrease in fracture stress and toughness noted in previous sections. Concurrently, the occurrence of the mechanical property minimum at a critical tilt angle of 75° rather than 90° suggests a complex interplay between defect geometry and lattice symmetry. Unlike the direct tensile loading at 90°, the 75° orientation creates a mixed-mode stress environment at the defect tips, where the applied tensile load is decomposed into both normal and shear components. The atomic rings in ψ-graphene are found to be highly sensitive to these combined forces.

To provide an objective quantitative evaluation of this localized environment and eliminate the ambiguities of manual region selection, a precise atom-sorting procedure was employed. For our simulation system (comprising 14,572 atoms, with 188 atoms removed for the *AR* = 4 defect), the critical zone at the sharp defect tips involves approximately 20 atoms. Therefore, the von Mises stress values of all individual atoms immediately prior to failure were sorted, and the top 20 highest-stressed atoms were selected. These critical atoms were found to naturally cluster at the sharp tips of the elliptical defect. Quantitative analysis reveals that the average von Mises stress within this top 20 critical atom group in the 75° model is 29.03 GPa, which is higher than that in the 90° configuration (26.59 GPa). Notably, the 75° configuration accumulates this much higher local stress at a lower global tensile strain, demonstrating that the combined tension-shear coupling induced by the 75° geometric tilt acts as an efficient stress concentrator. When this intense local force acts directly along the intrinsic weak bonding directions of the 5–6–7 rings, the sharp tips of the ellipse align effectively with these preferred cleavage pathways. This alignment directs the peak stress toward the weakest atomic bonds, driving their premature rupture and significantly accelerating crack initiation. This combined quantitative and geometric effect explains why the 75° orientation exhibits the lowest macroscopic mechanical strength.

## 4. Conclusions

In summary, the mechanical response and failure behavior of ψ-graphene containing elliptical nanoholes were systematically investigated through MD simulations. The main findings of this work are summarized below:

(1) Geometry and Loading Direction Dependency: The mechanical response of ψ-graphene is jointly determined by the defect geometry and the loading direction, exhibiting distinct anisotropic characteristics. Under *x*-axis loading, key mechanical properties such as fracture stress, strain, and toughness are severely compromised at an aspect ratio *AR* = 1 (a circular hole). Notably, a non-monotonic structural recovery is observed for elliptical defects under *x*-axial loading. By successfully decoupling the total vacancy fraction from the tip curvature via independent aspect ratio variations (*AR* = 1 to 8), we demonstrate that the elliptical defect can induce an effective structural blunting mechanism, enabling the defective structure to recover up to ∼80% of its pristine load-bearing capacity. In contrast, for loading along the *y*-axis, the material exhibits a substantial and persistent degradation in mechanical performance across all aspect ratios, showing relative insensitivity to the change in *AR*. This disparity highlights the complex interaction between defect geometry and the inherent anisotropy of the ψ-graphene lattice.
(2) The Influence of Defect Orientation: The orientation effect of the elliptical nanohole in ψ-graphene is markedly anisotropic; an anomalous mechanical minimum is identified at a critical defect tilt angle of *θ* = 75°, deviating from classical continuum mechanics predictions that favor a 90° minimum. Atomistic trajectories and quantitative computational analysis confirm that this phenomenon stems from a delicate competition between macro-stress fields and discrete lattice structure.

The results offer critical theoretical guidance for the structural design and reliability assessment of ψ-graphene-based materials, ensuring their stability in high-performance and extreme-condition applications.

## Figures and Tables

**Table 1:** Comparison of the calculated Young’s moduli for pristine ψ-graphene with established literature values.

Method	Ref.	Temperature (K)	$Y_{s}^{x}$ (N/m)	$Y_{s}^{y}$ (N/m)
AIREBO-M	This work	300	266.88	288.95
DFT	[9]	0	283.49	298.17
AIREBO-M	[12]	300	264.38	275.04
DFT	[11]	0	272.58	280.70

## Data Availability

The datasets generated during and/or analyzed during the current study are available from the corresponding author on reasonable request.

## Supplementary Materials

The following supporting information can be downloaded at: https://www.mdpi.com/article/10.3390/nano16181164/s1, Figure S1: Influence of the AIREBO-M potential cutoff radius (*r*_cutoff_) on the tensile behaviors of ψ-graphene. The tensile stress–strain profiles under *x*-axis loading with varying *r*_cutoff_ values from 2.0 Å to 2.25 Å are compared for: (a) the pristine nanosheet, and (b) the defective configuration with an aspect ratio of *AR* = 4; Figure S2: System size check for defective ψ-graphene systems under *x*-axis loading. The simulated stress–strain curves are compared between the middle-scale system (20.1 nm × 19.8 nm) and the expanded large-scale system (40.2 nm × 39.6 nm) for two representative defect geometries: (a) the circular nanohole (*AR* = 1), and (b) the elongated elliptical nanohole (*AR* = 4). The values in the legends denote the corresponding fracture strain and fracture stress.
