# Supplementary material for: Mechanical Response and Fracture Mechanisms of ψ-Graphene Containing Elliptical Defects: Effects of Aspect Ratio and Orientation
Source: Nanomaterials (Basel). 2026 Sep 16;16(18):1164. doi: 10.3390/nano16181164 (PMC13610704; doi:10.3390/nano16181164)
Supplement: Supplementary file 1 [file nanomaterials-16-01164-s001.zip › nanomaterials-4553141-supplementary.pdf]

nanomaterials
MDPI

*Supplementary Information*

# Mechanical Response and Fracture Mechanisms of ψ-Graphene Containing Elliptical Defects: Effects of Aspect Ratio and Orientation

**Xu Chang [1,*], Huichao Li [2,*] and Shuanghua Liang [1]**

[1] School of Computer Engineering, Jiangsu University of Architectural Technology, Xuzhou 221116, China; liangshuanghua@jsjzi.edu.cn
[2] School of Materials and Physics, China University of Mining and Technology, Xuzhou 221116, China
* Correspondence: 11051@jsviat.edu.cn (X.C.); hclee@cumt.edu.cn (H.L.)

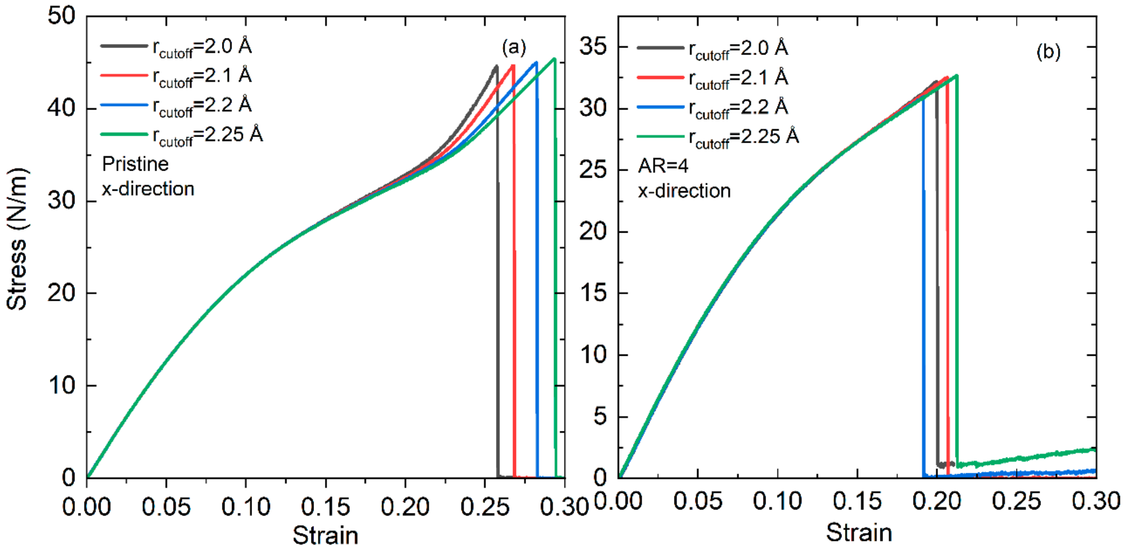


**Figure S1.** Influence of the AIREBO-M potential cutoff radius ($r_{cutoff}$) on the tensile behaviors of ψ-graphene. The tensile stress-strain profiles under $x$-axis loading with varying $r_{cutoff}$ values from 2.0 Å to 2.25 Å are compared for: (**a**) the pristine nanosheet, and (**b**) the defective configuration with an aspect ratio of $AR$=4.

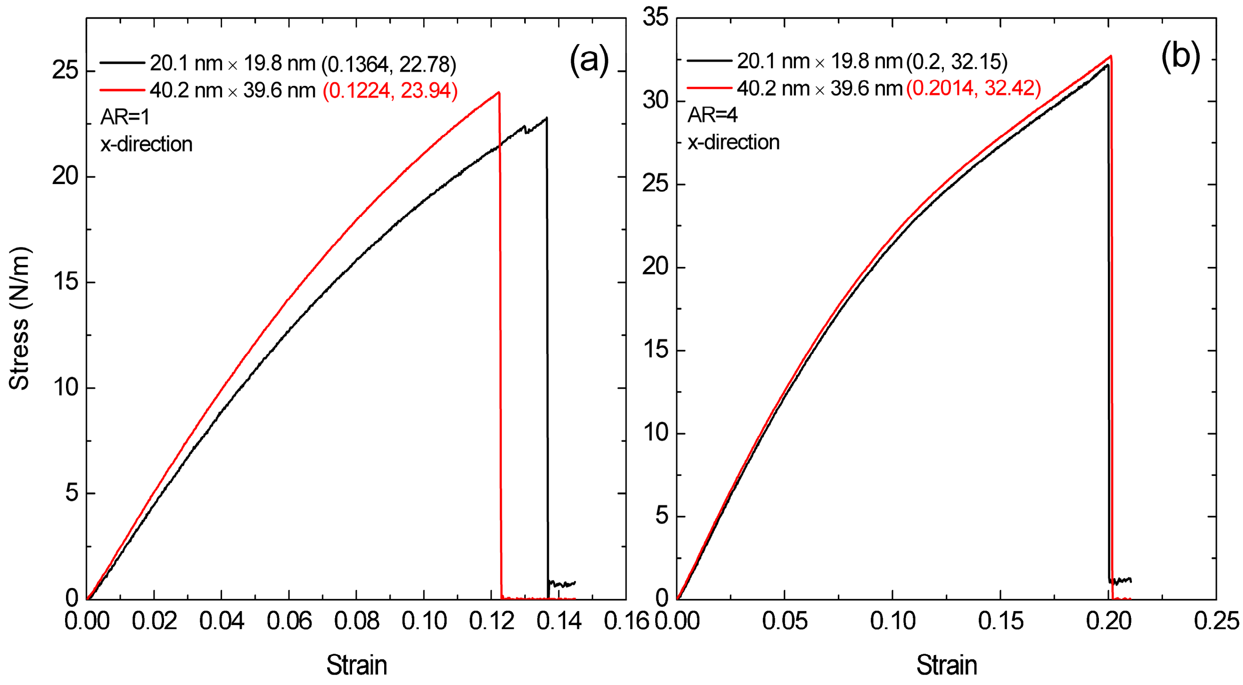


**Figure S2.** System size check for defective ψ-graphene systems under *x*-axis loading. The simulated stress-strain curves are compared between the middle-scale system (20.1 nm×19.8 nm) and the expanded large-scale system (40.2 nm×39.6 nm) for two representative defect geometries: (**a**) the circular nanohole (*AR*=1), and (**b**) the elongated elliptical nanohole (*AR*=4). The values in the legends denote the corresponding fracture strain and fracture stress.
